# Older Adults’ Perceptions of Smart City Initiatives to Age in Community

**DOI:** 10.1093/geroni/igab046.2143

**Published:** 2021-12-17

**Authors:** Jongwoong Kim

**Affiliations:** West Chester University of Pennsylvania, West Chester, Pennsylvania, United States

## Abstract

This project explores older American adults’ perceptions of smart city initiatives for them to “age in community” particularly in the northeast region. As the U.S. population is aging, it is imperative that the American cities can support their citizens to live in their preferred community environments for as long as they want. While there are many definitions of a smart city, some exemplary smarty city initiatives can be characterized as actively utilizing information and sensor technologies to promote efficiency and sustainability of city-wide systems, ultimately enhancing the quality of citizens' life. This project examines, in particular, seven smart city initiatives that are implemented globally: smart streetlights, health and fall monitoring system, community ridesharing, enhanced CCTVs, "age-friendly map," contact tracing app, and smart traffic system. By surveying those age 55 and older, with a representative sampling from the nine states in the northeast region, this project found that the vast majority of older Americans in this region would prefer to age in rural and suburban communities, and depending on where they prefer to age in (rural-exurban-suburban communities vs. urban-urban center communities) and gender (female vs. male), they perceive particular sets of smart city initiatives as more important for them to age in community. Furthermore, regardless of the community/location preference and demographic (gender, income level, and age) differences, 40% of the respondents expressed no concern of data or information privacy issues from these initiatives, opening some doors for the municipalities that plan to adopt some of these initiatives in the near future.

